# Annotation of epilepsy clinic letters for natural language processing

**DOI:** 10.1186/s13326-024-00316-z

**Published:** 2024-09-15

**Authors:** Beata Fonferko-Shadrach, Huw Strafford, Carys Jones, Russell A. Khan, Sharon Brown, Jenny Edwards, Jonathan Hawken, Luke E. Shrimpton, Catharine P. White, Robert Powell, Inder M. S. Sawhney, William O. Pickrell, Arron S. Lacey

**Affiliations:** 1 Swansea University Medical School, Swansea University, Swansea, Wales, UK; 2 Neurology Department, Swansea Bay University Health Board, Swansea, Wales, UK; 3 Paediatric Neurology Centre, Swansea Bay University Health Board, Swansea, Wales, UK

**Keywords:** Synthetic letters, Annotation guidelines, Gold standard, Natural language processing, Epilepsy

## Abstract

**Background:**

Natural language processing (NLP) is increasingly being used to extract structured information from unstructured text to assist clinical decision-making and aid healthcare research. The availability of expert-annotated documents for the development and validation of NLP applications is limited. We created synthetic clinical documents to address this, and to validate the Extraction of Epilepsy Clinical Text version 2 (ExECTv2) NLP pipeline.

**Methods:**

We created 200 synthetic clinic letters based on hospital outpatient consultations with epilepsy specialists. The letters were double annotated by trained clinicians and researchers according to agreed guidelines. We used the annotation tool, Markup, with an epilepsy concept list based on the Unified Medical Language System ontology. All annotations were reviewed, and a gold standard set of annotations was agreed and used to validate the performance of ExECTv2.

**Results:**

The overall inter-annotator agreement (IAA) between the two sets of annotations produced a per item F1 score of 0.73. Validating ExECTv2 using the gold standard gave an overall F1 score of 0.87 per item, and 0.90 per letter.

**Conclusion:**

The synthetic letters, annotations, and annotation guidelines have been made freely available. To our knowledge, this is the first publicly available set of annotated epilepsy clinic letters and guidelines that can be used for NLP researchers with minimum epilepsy knowledge. The IAA results show that clinical text annotation tasks are difficult and require a gold standard to be arranged by researcher consensus. The results for ExECTv2, our automated epilepsy NLP pipeline, extracted detailed epilepsy information from unstructured epilepsy letters with more accuracy than human annotators, further confirming the utility of NLP for clinical and research applications.

**Supplementary Information:**

The online version contains supplementary material available at 10.1186/s13326-024-00316-z.

## Introduction

Natural language processing (NLP) applications are being developed for use in healthcare and health research [1]. NLP systems can extract structured information from unstructured clinic text at scale, to aid clinical decision making and to provide structured research data [2]. For example in epilepsy, one of the most common neurological conditions, NLP has been used to: extract risk factors for sudden death [3], analyse long-term seizure freedom patterns [4], and identify epilepsy surgery candidates [5].

The development and validation of NLP applications depends on the availability of expert-annotated documents [6–8]. A few deidentified corpora have been created for specific applications, for example i2b2: Informatics for Integrating Biology and the Bedside and n2c2 NLP Research datasets [9] or CLEF eHealth Task 2013 Dataset [10]. However, in general, there is a lack of freely available clinical documents for NLP development, due mainly to constraints around patient identifiable data. For epilepsy specific applications attention has been given to distinct concept extraction, with researchers requiring access to patient notes and choosing various annotation approaches [11–13]. To our knowledge there are no published epilepsy clinical text annotations and annotation guidelines for the extraction of a full range of epilepsy concepts and relations.

Epilepsy clinic letters are a record of an individual’s consultation with an epilepsy specialist such as a neurologist or specialist nurse. They describe medical history, seizures and their frequency as reported during the consultation, test results, treatment, and diagnosis. Over time they form a detailed record of an individual’s epilepsy, changes in seizure frequency, diagnostic clarification, and the effect of treatment. They hold a vast amount of information that can be used in a clinical setting or in population-wide research.

We aimed to create a set of realistic, synthetic epilepsy clinic letters and annotation guidelines covering the contents of typical epilepsy clinic consultations to assist in information extraction application development. We used the synthetic letters to benchmark the performance of version 2 of our NLP Extraction of Epilepsy Clinical Text (ExECTv2) pipeline [14].

## Method

### Synthetic letters

We produced 200 synthetic epilepsy clinic letters, based on United Kingdom (UK) hospital outpatient epilepsy clinic consultations. Epilepsy clinic letters are written by clinicians and describe relevant details, discussions, investigations, and management plans. They are part of the patient health record and are written in a variety of styles, lengths, and formats.

The synthetic letters were written by neurology consultants, specialist trainees, and epilepsy specialist nurses to ensure a variation in writing styles and content. They were based on real clinic letters but contained completely synthetic information and any patient or clinician information in the letters is completely fictitious, i.e. no real personal, demographic, or clinical information is included. Four letters were duplicated within the set to test for consistency in annotations.

### Annotations

The letters were double annotated by four trained researchers and clinicians (100 letters each) according to annotation guidelines formed during the development of ExECT. We developed the annotation guidelines based on previous annotation sessions and modified them to incorporate annotators’ suggestions, providing examples derived from clinic letters to assist with more difficult cases.

**Fig. 1:**
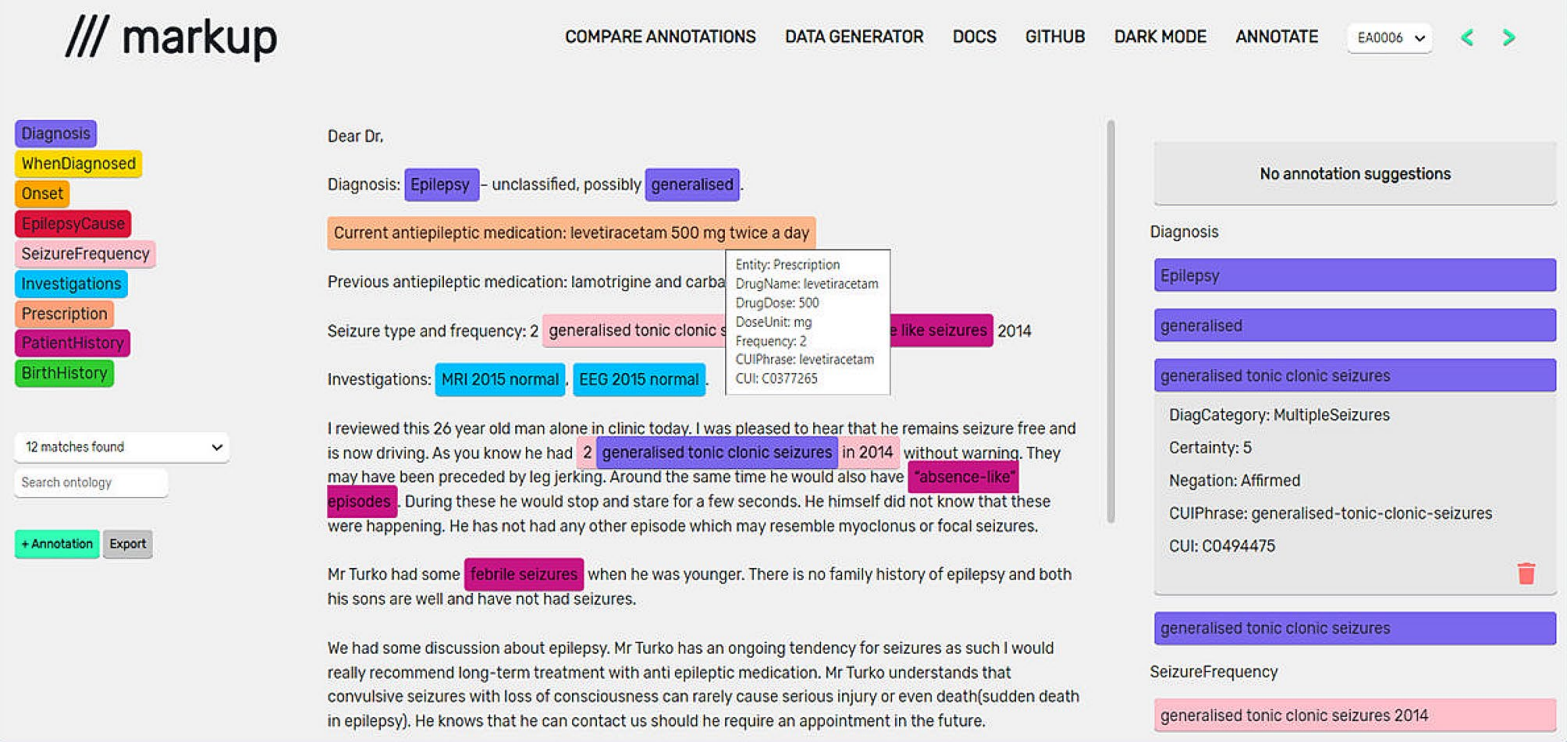
Annotating synthetic letters in Markup (www.getmarkup.com). Annotation types are listed on the left-hand side, above the UMLS selection dropdown. Completed annotations are listed on the right-hand side

We used the annotation tool, Markup [15] with an epilepsy concept list based on the Unified Medical Language System (UMLS) ontology [16] with mapping of terms from the International League Against Epilepsy (ILAE) epilepsy and seizure classification [17, 18]. Markup provides annotators with a list of entities (concepts) to be annotated and drop-down lists of features (attributes to be assigned to each entity, including UMLS concept unique identifiers [CUIs]) associated with each diagnostic or treatment term (Fig. 1). We ran several trial sessions to ensure familiarity with Markup and the annotation process before the annotation task.

Entities that were annotated included:

#### Birth history

birth age, perinatal events, normal/abnormal birth;

#### Diagnosis

epilepsy, epilepsy type/syndrome, seizure type;

#### Epilepsy cause

clear statements identifying past events or comorbidities causing an individual’s epilepsy;

#### Investigations

EEG (including examination type), CT, and MRI results, annotated as normal, abnormal, or not stated;

#### Onset

time of onset of epilepsy or specific seizure types, expressed as age, date, or time since first epileptic seizure or mention of epilepsy;

#### Patient history

unspecified seizures (seizures, blank episodes), febrile seizures, major health events, and comorbidities, with age, date, or time since/onset of the event;

#### Prescriptions

current prescribed antiseizure medications (ASM) with dose, dose unit, and frequency;

#### Seizure frequency

number of seizures, by type if stated (including periods of seizure freedom) since or during specific point in time/time period/date, or changes in seizure frequency since/during specified time or since last clinic visit;

#### When diagnosed

age, date, or time since the diagnosis of epilepsy.

Levels of certainty expressed in the statements, ranging from 1 (negation) to 5 (strong affirmation) were assigned to phrases relating to diagnosis and patient history (Supplementary Table 1).

### Inter-annotator agreement

We combined the annotation sets from all four annotators, creating two sets of 200 annotations each. We compared these two sets (of 200 letters each) using inter-annotator agreement (IAA). IAA, which assesses the level of agreement between the annotators, was calculated using F1 score, the harmonic mean of precision (positive predictive value) and recall (sensitivity), an established information retrieval performance measure [19]. We define agreement when two annotators select the same entity and attributes for a specific term. All annotations were reviewed during consensus meetings.

The final corrected set, representing consensus opinion, formed the gold standard which we used to validate ExECTv2, with the IAA scores providing a benchmark measure for the pipeline’s performance [20]. ExECT is an epilepsy NLP pipeline written within GATE (Generalised Architecture for Text Engineering). See supplementary information for a figure detailing the ExECT pipeline (Supplementary Fig. 1) and Fonferko-Shadrach et al. for further details on ExECT [14]. ExECTv2 has several improvements over version 1 which include: an expanded range of extracted terms, updated gazetteers that include the most recent International League Against Epilepsy (ILAE) classification system, and added rules for combined seizure and epilepsy terms [21]. We used R version 4.1.0 to calculate per item (every mention of the entity) and per letter (correct extraction of the term in a letter) validation scores.

## Results

The 200 synthetic letters, annotations, and annotation guidelines are available on Zenodo (10.5281/zenodo.8381079, annotations in JSON format: 10.5281/zenodo.8356493 and 10.5281/zenodo.8382588).

The overall F1 score for human IAA was 0.73. The scores for specific entities varied, with the lowest result for *When Diagnosed* (0.45) and the highest for *Prescriptions* (0.87), Table 1. Validation of ExECTv2 against the gold standard produced an overall per item (per annotation) F1 score of 0.87, with *Seizure Frequency* having the lowest result (0.66) and *Birth History* the highest (0.97). There was less variation between the scores for different entities. Per letter results are more uniform, with an overall F1 score of 0.90, with *Seizure Frequency* still having the lowest score (0.68), Table 1, (full results in Supplementary Table 2).

For broader categories, such as *Patient History*, for which multiple mentions of unspecified seizures may be captured, additional validation was produced, excluding unspecified seizures. For example, F1 score for comorbidities (including history of febrile seizures and dissociative seizures) was 0.86 per item and 0.89 per letter.

**Table 1:** Inter annotator agreement (IAA) for 200 synthetic letters, performed in Markup. All features excluding Concept Unique Identifiers (CUIs) and validation of extraction of Epilepsy Clinical text (ExECT)v2 pipeline against the gold standard set of annotations with all features. Per item scores (every mention of the entity) and per letter (at least one correct extraction of the entity with features in a letter)

	IAA (human annotators)	Validation* of ExECT v2 against the gold standard			
Annotation	F1 score	Number of annotations in the gold standard	Per item F1 score		Per letter F1 score
Birth History	0.69	47	0.97		0.98
Diagnosis**	0.83	572	0.85		0.94
Epilepsy Cause	0.67	36	0.90		0.92
Investigations	0.82	183	0.95		0.95
Onset	0.61	22	0.96		0.95
Patient History†	0.57	620	0.78		0.89
Prescription	0.87	290	0.87		0.87
Seizure Frequency	0.47	260	0.66		0.68
When Diagnosed	0.45	17	0.91		0.91
All‡	0.73	2047	0.87		0.90

* Annotations with features including certainty for Diagnosis and Patient History only

** Includes a feature distinguishing whether based on epilepsy, multiple seizures, or a single seizure. Per letter validation was based on epilepsy or multiple seizure annotations of certainty level of 4 (probable) and 5 (definite) and matched by CUI i.e. at least one correctly matched epilepsy or seizure diagnosis of specific type. With the epilepsy / seizure type ignored we can match for at least one correct annotation of epilepsy (based on epilepsy or multiple seizures) of certainty level 4 or 5, and this gives F1 score 0.99

† Includes Negation to identify negated history of febrile seizures

‡ Average of all documents scores

## Discussion

We have created and annotated synthetic epilepsy clinical documents, making them available for the epilepsy research community. We have shown that the performance of an automated information extraction pipeline (ExECT) exceeds annotations created by humans.

Our results show that identifying and classifying entities can be hard for annotators. The main errors observed in our test arose from missing annotations and attributes, or misclassification of concepts, for example annotating unspecified seizures under epilepsy diagnosis. Missing or misassigned CUIs were also common. As this did not reflect annotators’ choice but occurred in error, CUIs were disregarded from the IAA (annotations were compared on the phrase selection/classification and attributes).

The range of features to be assigned and the need for matching against the UMLS list, reflecting the complexity of the rule-based system used in ExECT, may have contributed to annotator fatigue and subsequent errors. More structured entities, for example prescriptions, are easier to annotate than items which ‘relay a story’ given by patients during consultation, as in seizure frequency or patient history [22]. Seizure frequency for example is recorded in a wide variety of formats and styles and often there are references to frequencies of multiple different seizure or event types. This reflects the real-world difficulty in recording seizures frequency. This difficulty with very unstructured or variable text is a significant disadvantage of annotating text for a rule-based system as compared to classifying phrases for a machine learning model [4]. Detailed clear guidelines developed in collaboration with annotators and annotation trials reduce errors [23].

The choice of items identified for annotation, although wide, does not include all concepts present in epilepsy documents (e.g. seizure semiology, technical details of investigation results such as EEGs, and family history) or negated statements. Also ExECT does not currently extract all epilepsy concepts. This is a limitation of this annotation set. However, used as a guide, the annotations can be expanded or limited to fewer entities.

The gold standard set of annotations was reached through discussion and consent regarding error correction (annotation/feature reassignment, missing CUI allocation). It is important to note that variation in structure, writing styles, and content across documents from different sources makes it necessary for each application to be validated when used on different corpora. For example, for our work on linking seizure frequency and genetic data, validation was performed on 100 deidentified real epilepsy clinic letters, producing F1 scores of 0.69 per item and 0.88 per letter [24]. The validation against the gold standard based on the synthetic letters produced slightly lower results.

It is difficult to compare these results to the validation of ExECTv1, which had fewer annotation types and features, the overall scores per item and per letter are however similar. As the term matching was performed using CUIs these results suggest an improvement from the original pipeline.

## Conclusions

We have made the 200 synthetic letters, the annotations, and the annotation guidelines freely available. To our knowledge, this is the first publicly available set of annotated epilepsy clinic letters and guidelines that can be used for NLP researchers with minimum epilepsy knowledge. The IAA results show that the clinical text annotation tasks can be difficult, with a need for a gold standard to be arranged by researcher consensus. The performance of ExECTv2 was better than the agreement reached by the annotators. Finally, we note that the synthetic letters may be used to train large language models which might be the way forward to obtain greater number of documents for applications’ development.

## Electronic supplementary material

Below is the link to the electronic supplementary material.

Supplementary Material 1

## Data Availability

Synthetic epilepsy clinic letters and annotations are available on Zenodo, https://zenodo.org/records/8381080, Annotations in JSON format, https://zenodo.org/records/8356494.Annotation guidelines are available at https://zenodo.org/records/8382589Markup annotation outputs (human annotations) for each annotation type as CSV format files are available at https://zenodo.org/records/12520180.

## Abbreviations

ASM: Antiseizure medications
CUI: Concept unique identifier
ExECT: Extraction of Epilepsy Clinical Text
GATE: Generalised Architecture for Text Engineering
IAA: Inter—annotator agreement
ILAE: International League Against Epilepsy
NLP: Natural language processing
UMLS: Unified Medical Language System
