# Supplementary material for: Annotation of epilepsy clinic letters for natural language processing
Source: J Biomed Semantics. 2024 Sep 15;15:17. doi: 10.1186/s13326-024-00316-z (PMC11402197; doi:10.1186/s13326-024-00316-z)
Supplement: Supplementary file 1 — Supplementary Material 1 [file 13326_2024_316_MOESM1_ESM.docx]

**Supplementary Information**

| Certainty Level | Description | Example |
| --- | --- | --- |
| 1 | Negation | This patient does not have epilepsy |
| 2 | Unlikely | It is unlikely that this man has epilepsy |
| 3 | Possible | I think epilepsy is a possibility |
| 4 | Probable | Based on the description of events I think she probably does have temporal lobe epilepsy |
| 5 | Definite | Diagnosis: Juvenile Myoclonic Epilepsy |

**Supplementary Table 1**: Certainty levels, descriptions, and examples.

|  | **Validation***  **Per item** | | | **Validation***  **Per letter** | | |
| --- | --- | --- | --- | --- | --- | --- |
| Annotation | **Precision** | **Recall** | **F1 Score** | **Precision** | **Recall** | **F1 Score** |
| Birth History | 1.00 | 0.94 | 0.97 | 1.00 | 0.96 | 0.98 |
| Diagnosis** | 0.86 | 0.85 | 0.85 | 0.95 | 0.92 | 0.94 |
|  |  |  |  |  |  |  |
| Epilepsy Cause | 0.97 | 0.83 | 0.90 | 0.97 | 0.88 | 0.92 |
| Investigations | 0.96 | 0.94 | 0.95 | 0.96 | 0.94 | 0.95 |
| Onset | 1.00 | 0.92 | 0.96 | 1.00 | 0.91 | 0.95 |
| Patient History† | 0.83 | 0.73 | 0.78 | 0.93 | 0.85 | 0.89 |
| Prescription | 0.92 | 0.82 | 0.87 | 0.92 | 0.83 | 0.87 |
| Seizure Frequency | 0.72 | 0.61 | 0.66 | 0.74 | 0.64 | 0.68 |
| When Diagnosed | 0.94 | 0.88 | 0.91 | 0.94 | 0.88 | 0.91 |
|  |  |  |  |  |  |  |
| All‡ | 0.91 | 0.84 | 0.87 | 0.93 | 0.87 | 0.90 |

**Supplementary Table 2**: ExECTv2 validation against the synthetic letter gold standard set of annotations – full results. Precision (equivalent of positive predictive value) is a ratio of correctly retrieved annotations to all those retrieved, recall (equivalent of sensitivity) is a ratio of correctly retrieved annotations to all annotations (as identified by the gold standard), F1 score is the harmonic mean of precision and recall that gives a single measure of the system’s performance and ranges from 0 to 1.

* Annotations with features including certainty for Diagnosis and Patient History only.

** Includes a feature distinguishing whether based on epilepsy, multiple seizures, or a single seizure. Per letter validation was based on epilepsy or multiple seizure annotations of certainty level of 4 (probable) and 5 (definite) and matched by CUI i.e. at least one correctly matched epilepsy or seizure diagnosis of specific type.

† Includes Negation to identify negated history of febrile seizures.

‡ Average of all documents scores.


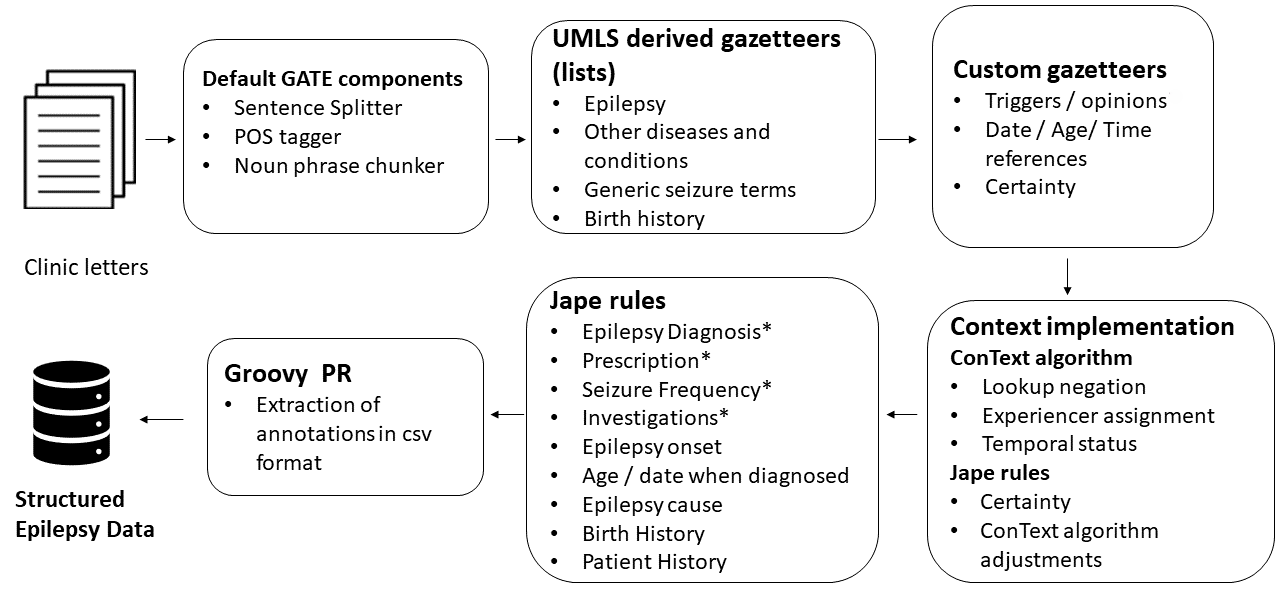


**Supplementary Figure1**: Extraction of Epilepsy Clinical Text (ExECT) version2 based on General Architecture for Text Engeenering (GATE).

* Annotations extracted in ExECT version1.
